# Integrating network pharmacology, molecular docking, and experimental validation, this study explores the mechanism of action of Typhonii Rhizoma in colon cancer

**DOI:** 10.3389/fonc.2026.1815506

**Published:** 2026-05-07

**Authors:** Fei-Yan Zhou, Bo-Hong Xu, Yue Du, Han Yu, Fang-Hua Song

**Affiliations:** 1 Department of Oncology, Xinhua Hospital Affiliated to Dalian University, Dalian, Liaoning, China; 2 Center of Pelvic Floor, Xinhua Hospital Affiliated to Dalian University, Dalian, Liaoning, China

**Keywords:** colon cancer, experimental verification, molecular docking, network pharmacology, Typhonii Rhizoma

## Abstract

**Introduction:**

Typhonii Rhizoma (TR), the dried tuber of *Typhonium giganteum* Engl. from the Araceae family, exhibits significant anticancer activity, although its underlying mechanism in colon cancer remains unclear.

**Methods:**

This study employed a comprehensive approach combining network pharmacology, molecular docking, and experimental validation to elucidate the potential anticancer mechanism of TR against colon cancer. Initially, the targets of TR and colon cancer were retrieved from public databases, and their intersection targets were identified. Subsequently, these targets were subjected to protein-protein interaction (PPI) network analysis, Gene Ontology (GO), and Kyoto Encyclopedia of Genes and Genomes (KEGG) pathway enrichment analyses to identify key pathways. Molecular docking was then performed to evaluate potential interactions between active TR components and core targets. Finally, the predicted mechanisms were validated through a series of *in vitro* experiments.

**Results:**

Network pharmacology analysis identified 101 intersection targets shared by TR and colon cancer, with the PI3K/AKT signaling pathway emerging as the core pathway mediating the anti-colon cancer effects of TR. Furthermore, molecular docking suggested favorable interactions between several candidate components of TR and AKT1/PI3K. Consistent with these predictions, *in vitro* experiments demonstrated that TR was associated with reduced PI3K/AKT pathway activity and suppressed cell proliferation.

**Discussion:**

These findings suggest that TR inhibits malignant phenotypes of colon cancer cells and may be associated with modulation of the PI3K/AKT pathway *in vitro*. This study provides preliminary mechanistic support for further investigation of TR in colon cancer and a rationale for future *in vivo* and rational combination studies.

## Highlights

- Explored the mechanism of TR against colon cancer via network pharmacology and validation.
- Identified PI3K/AKT signaling pathway as the key pathway of TR.
- Suggested potential interactions between candidate TR components and AKT1/PI3K, alongside inhibition of colon cancer cell proliferation.

## 1 Introduction

Colorectal cancer is the third most common and second deadliest malignancy worldwide, with more than 1.85 million new cases and 850, 000 deaths annually (1). To date, colorectal cancer has become one of the most significant challenges facing health systems in many countries (2–4). Despite significant advances in diagnostic techniques (such as advanced colonoscopy, imaging, and molecular marker detection) and treatments (including surgery, radiotherapy, chemotherapy, targeted therapy, and immunotherapy) in recent years (5–8). The prognosis for patients with advanced or metastatic colorectal cancer (mCRC) remains grim, with a significantly lower 5-year survival rate than that for patients with early-stage cancer (9, 10). A major challenge in the treatment of colon cancer is the heterogeneity of the tumor microenvironment, which includes cancer cells, stromal cells, immune cells, and extracellular matrix components. This complexity often limits the efficacy of conventional therapies, thus necessitating the development of more specific, multi-targeted treatment strategies (11, 12). Furthermore, resistance to chemotherapy and targeted drugs remains a critical issue, making the need for alternative natural therapies even more urgent.

Typhonii Rhizoma (TR) is the dried tuber of *Typhonium giganteum* Engl., a plant in the Araceae family (13). Typhonii Rhizoma occupies a place among the Chinese medicinal products listed in the Chinese Pharmacopoeia. Its rhizome contains a variety of active ingredients, including polysaccharides, cerebrosides, glycosides, riboflavin, and alkaloids (14). These active ingredients endow Typhonii Rhizoma with many beneficial effects. It is excellent in neuroprotection, with varying degrees of sedation, anticonvulsant, and analgesic effects (15). It also has significant anti-inflammatory properties (16). Of particular interest is its anti-tumor effect. In fact, the anti-tumor efficacy of Typhonii Rhizoma extract has been confirmed in many studies. For example, a study by Gu et al. in 2018 showed that two trace alkaloids, berberine (BER) and coptisine (COP), from the n-butanol extract of Typhonii Rhizoma, could induce cell cycle arrest in a concentration-dependent manner *in vitro* and effectively reduce the viability of human glioma U87 cells (17). However, to date, the structures of these active ingredients have not been thoroughly studied and characterized, and it is unclear whether they play a key role in promoting the antitumor effect of Typhonii Rhizoma.

Network pharmacology (NP) is a new discipline based on systems biology theory, biological system network analysis, multi-target drug molecule design, and specific signal node selection (18–21). It provides a new methodological perspective for understanding traditional medicine from a holistic perspective (22). In addition, with the help of mapping biological networks provides profound insights into biomolecular interactions and enhances our understanding of drug mechanisms, multipharmacology, and disease etiolog (23). Molecular docking technology is a research method used for drug discovery and screening. By screening ligand and receptor structures in a database and combining them with algorithms in software, the most suitable binding conformations and combinations of ligands and receptors can be found (24–27). Network pharmacology and molecular docking have been widely used to accurately elucidate the pharmacological activity of potential therapeutic drugs.

**Figure 1:**
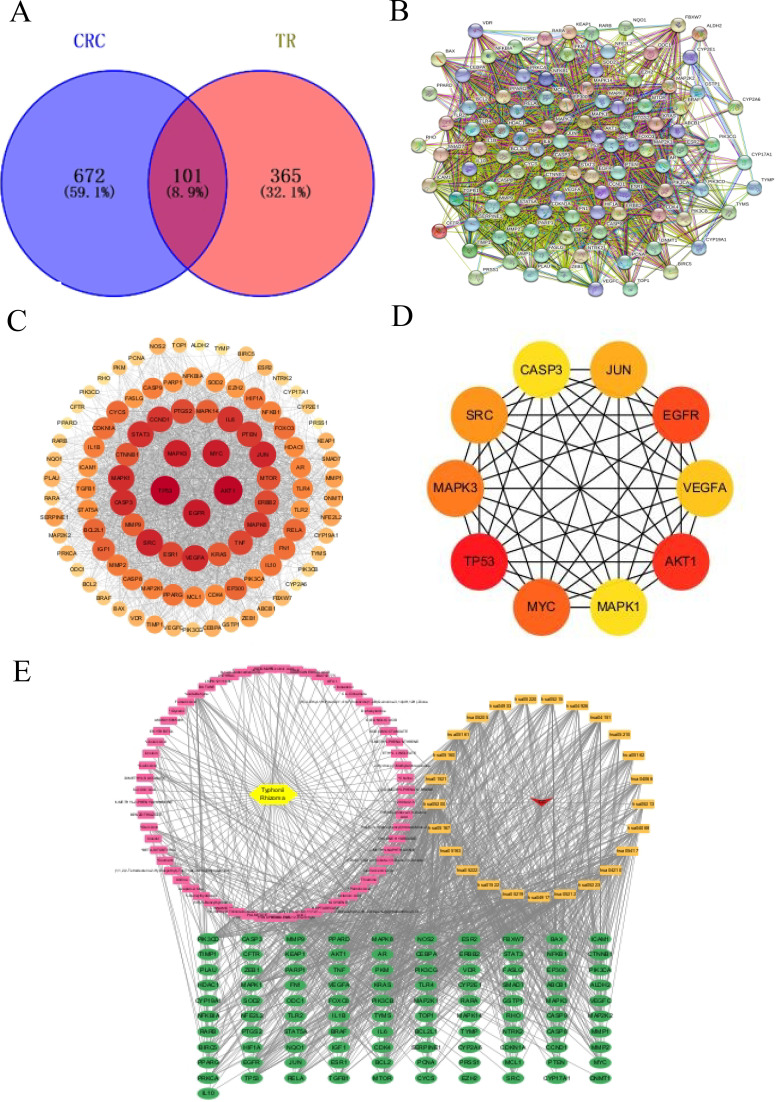
Screening and network construction of potential targets of Typhonii Rhizoma against colon cancer. **(A)** Venn diagram showing the overlapping targets between Typhonii Rhizoma and colon cancer. **(B, C)** PPI network of the overlapping targets constructed using STRING and visualized in Cytoscape. **(D)** Hub genes identified from the PPI network using the CytoHubba plugin. **(E)** Disease-pathway-target-component-drug network constructed in Cytoscape. Pink rectangles represent active compound components, yellow hexagons represent compounds, red inverted triangles represent disease, orange rectangles represent enriched pathways, green ellipses represent overlapping target genes, and lines indicate the interactions among components, targets, pathways, and disease.

In this study, we employed a combination of network pharmacology, molecular docking, and experimental methods to demonstrate the mechanism of action of Typhonii Rhizoma in the treatment of Colon Cancer (CC). This study provides preliminary mechanistic support for further investigation of Typhonii Rhizoma in colon cancer. **Figure 1** summarizes the target screening and network construction process used in this study.

## 2 Methods and materials

### 2.1 Reagents

The experiment involves experimental reagents (**Table 1**).

**Table 1:** Experiment-related reagents.

Experimental reagents	Manufacturer	Experimental reagents	Manufacturer
MEM medium	Servicebio	AKT1 Antibody	Biodragon
DMEM Medium	Servicebio	p-AKT1 Antibody	Biodragon
Penicillin-Streptomycin Mixture	Servicebio	PI3K Antibody	Biodragon
Fetal bovine serum	BBI	p-PI3K Antibody	Biodragon
0.25% trypsin	EallBio	HRP-labeled goat anti-rabbit IgG	Servicebio
0.25% Trypsin (EDTA-free)	Servicebio	β-actin Polyclonal Antibody	Biodragon
GAPDH Antibody	Biodragon	RIPA Buffer/Cell Lysate	Solarbio
Cell Counting Kit-8	Servicebio	Protease Inhibitor Mixture	Epizyme
Ready-to-Use BCA Protein Quantification Kit	Epizyme	Phosphatase Inhibitor Mixture	Epizyme
5× Protein Loading Buffer (DTT-containing)	Epizyme	4% Hematoxylin and Eosin Fixative	Solarbio
15% One-Step PAGE Gel Rapid Preparation Kit	Epizyme	Annexin V-FITC/PI Apoptosis Detection Kit	Elabscience
10% One-Step PAGE Gel Rapid Preparation Kit	Epizyme	Apoptosis and Cell Cycle Detection Kit	Servicebio
PMSF	Epizyme	Crystal violet reagent	Solarbio
Universal Antibody Dilution Buffer	Biodragon	4% polyformaldehyde	Solarbio
WesternBright ECL	Biodragon	Matrigel	ABW
Western Blot Membrane Regeneration Solution	Solarbio	electrophoresis buffer	Genefist
Dimethyl sulfoxide	Solarbio	20× Rapid Blotting Buffer	NCM
Rabbit Anti-Bcl-2 Monoclonal Antibody	Bioss	10×TBST	Solarbio
Bax Rabbit mAb	Biodragon	PVDF membrane	Solarbio

### 2.2 Prediction of colon cancer and targets of Typhonii Rhizoma

All databases were queried on July 10, 2025. Colon cancer-related targets were collected from the OMIM (28) and GeneCards (29) databases using the search terms “colon cancer” and “Homo sapiens”, and only human genes/proteins were retained for further analysis. Typhonii Rhizoma-related targets were retrieved from the BATMAN-TCM (30) and HERB (31) databases using the keyword “Typhonii Rhizoma”. In this study, herb-related targets were obtained directly from BATMAN-TCM and HERB at the herb-target level, rather than through initial monomer-compound screening in TCMSP; therefore, no oral bioavailability (OB) or drug-likeness (DL)-based compound prefilter was applied at the initial target collection step. Targets collected from the above databases were merged into a single candidate list, duplicate entries were removed, and protein/target names were converted into official gene symbols using the UniProt database. The standardized colon cancer-related and TR-related target sets were then intersected to obtain the potential targets of TR against colon cancer.

### 2.3 Screening of drug-disease co-targets and construction of PPI network

The standardized gene sets obtained in Section 2.2 were imported into Venny 2.1.0 (https://bioinfogp.cnb.csic.es/tools/venny/index.html) to identify the overlapping targets between Typhonii Rhizoma and colon cancer. These intersecting genes were considered potential therapeutic targets of TR against colon cancer. The overlapping targets were then imported into the STRING database (https://cn.string-db.org/) to construct the protein-protein interaction (PPI) network, with the organism restricted to Homo sapiens and the minimum required interaction score set at 0.400 (32). The resulting TSV file was imported into Cytoscape 3.10.3 for visualization, and hub genes were identified using the CytoHubba plugin (26).

### 2.4 GO and KEGG enrichment analysis

GO and KEGG enrichment analysis of potential targets of Typhonii Rhizoma for colon cancer treatment was performed using the “Functional Annotation” feature on the DAVID website (https://davidbioinformatics.nih.gov/) (33). The obtained data were organized and visualized using bioinformatics (http://www.bioinformatics.com.cn/).

### 2.5 Construction of disease-pathway-target-component-drug network

A disease-pathway-target-component-drug network was constructed by introducing Typhonii Rhizoma, its components, potential targets for colon cancer treatment, the KEGG pathway, and colon cancer into Cytoscape. Nodes represent Typhonii Rhizoma, drug components, genes, pathways, and colon cancer, respectively, while connecting lines represent relationships between biomolecules.

### 2.6 External validation of core targets

#### 2.6.1 Expression level of core target genes

In GEPIA’s “Expression DIY” (http://gepia.cancer-pku.cn/), the mRNA expression level of the core target and the pathological stage were verified (34). |Log2FC| Cutoff: 0.5, p-value cutoff: 0.05.

#### 2.6.2 Expression levels of core target proteins

To investigate the expression of core targets in colon cancer tissue, we analyzed core targets in the Human Protein Atlas database (https://www.proteinatlas.org/) (35). We compared the protein expression levels of core targets in colon cancer tissue and normal colon tissue.

#### 2.6.3 Prognostic analysis of hub genes

To further reduce target-selection bias and assess the potential clinical relevance of hub genes, prognostic analysis was performed for the top 10 hub genes identified from the PPI network using publicly available colon cancer datasets in Sangerbox 3.0(http://sangerbox.com/home.html). Overall survival (OS) was evaluated in the TCGA-COAD cohort. Patients were stratified into high- and low-expression groups according to the median expression level of each gene. Kaplan–Meier survival curves were compared using the log-rank test, and hazard ratios (HRs) with 95% confidence intervals (CIs) were recorded when available. These analyses were used as supportive evidence to examine the prognostic heterogeneity of hub genes rather than as the sole basis for target selection.

### 2.7 Molecular docking

The CytoNCA (36) plugin of Cytoscape software was used to screen out the five active ingredients with the highest Degree values in the “traditional Chinese medicine-active ingredient-target-disease-pathway” network diagram and perform molecular docking with the ten core proteins with the highest Degree values in the PPI network. The structures of the compounds were obtained from the TCMSP database (https://tcmsp-e.com/index.php) (37), and the protein structures were obtained from the PDB database (https://www.rcsb.org/) (38). The intermolecular interactions between the main components of Typhonii Rhizoma and the core targets identified in this study are predicted by predicting the binding mode and affinity. The ten core targets were designated as receptors, and the five active ingredients were used as ligands for docking verification. The mol2 format files of the five active ingredients of Typhonii Rhizoma were retrieved from the TCMSP database, and the protein data (PDB) files of the core target proteins were downloaded using the UniProt ID in the PDB database. CB-Dock2 (39) was used to pretreat small molecule ligands and target proteins, including energy optimization, hydrogenation, removal of water molecules, and energy minimization. The docking site with the lowest Vina score was selected as the optimal binding mode. Subsequently, five active ingredients were molecularly docked with key proteins in the PI3K-AKT pathway, and the binding energy was calculated. The binding mode was then visualized in 3D.

### 2.8 Preparation of Typhonii Rhizoma extract

Typhonii Rhizoma ethanol extract powder was purchased from Nanjing Daosufu Biotechnology Co., Ltd. A total of 0.2 g of extract powder was accurately weighed and completely dissolved in 1 mL of sterile dimethyl sulfoxide (DMSO) to prepare a stock solution at 200 mg/mL. The solution was vortexed at 1500 rpm for 10 min until fully dissolved, aliquoted, and stored at −20 °C protected from light. Before each experiment, the stock solution was thawed and incubated in a 37 °C water bath for 10 min. Working solutions were prepared by serial dilution with culture medium, with the highest final working concentration set at 5 mg/mL. All preparation and dilution procedures were performed under aseptic conditions. Because a crude extract rather than a purified monomer was used in this study, the treatment concentrations were expressed as mass concentration (mg/mL). The final DMSO concentration in each working solution was maintained below 0.25%, and the vehicle control group received the corresponding concentration of DMSO without extract. No pathway-selective pharmacologic comparator (such as a PI3K or AKT inhibitor) was included in the present study.

### 2.9 Cell culture

The Caco-2 and HCA-7 human colon cancer cell lines were purchased from Ausys Biotechnology (Shanghai) Co., Ltd., and cultured in MEM and DMEM complete medium, respectively.

### 2.10 Cell viability assay

5×10^4^ cells per well in 96-well plates containing MEM/DMEM medium. After incubation for 24 hours, the cells were treated with fresh MEM/DMEM medium containing different concentrations of TR (0, 1, 2, 3, 4, 5 mg/mL) for another 24 hours. At the end of the treatment, the medium was replaced with fresh MEM/DMEM containing 10 μL CCK-8 reagent and incubated for 1 hour. Finally, the absorbance of the entire plate was measured at 450 nm using a full-wavelength microplate reader (BioTek, United States). Each treatment group was repeated 5 times, and the experiment was repeated 3 times.

### 2.11 Scratch test

Cells were seeded in 6-well cell culture plates and grown to 90% confluence. Cell monolayer scratches were manually created using a 200 μL pipette tip. Cells were washed three times with PBS to remove excess cells. Different concentrations of TR (0, 1, 2, 3, 4, 5 mg/mL) were added to the plates. Scratch images were captured using a microscope (NOVEL, Ningbo Yongxin Optics) at 0 and 24 hours after TR treatment. Image processing was performed using ImageJ software with a scratch healing analysis plugin. The migration rate was calculated using the formula: Migration Rate(%)= [(A_0_-A_24_)/A_0_]×100, where A_0_ represents the initial scratch area (0h), and A_24_ represents the scratch area after treatment (24h). All data were verified through three independent experiments.

### 2.12 Transwell invasion experiment

Caco-2 and HCA-7 cells (4×10^4^ cells/well) were cultured in the upper chamber of a 24-well plate using serum-free MEM and DMEM media, respectively, while the lower chamber was filled with 500 μL of complete culture medium. Cells were treated with the indicated concentrations of TR extract (0, 2, 4mg/mL) for 24 h. Subsequently, these chambers were fixed with 4% paraformaldehyde and stained with 0.1% crystal violet. After washing with PBS, the chambers were allowed to air dry, and images of the invasion were captured using a microscope (NOVEL, Ningbo Yongxin Optics).

### 2.13 Flow cytometry analysis

Caco-2 and HCA-7 cells treated with 2 mg/mL and 4 mg/mL TR were collected using EDTA-free trypsin (KeyGEN BioTECH, Nanjing, China). After centrifugation with phosphate-buffered saline (PBS), 1 ml of pre-chilled 70% ethanol was added, and the cells were mixed by pipetting and then fixed overnight at 4 °C. The next day, the cells were removed, centrifuged, and the supernatant was discarded. The cells were resuspended in PBS and centrifuged again. 500μl of the prepared staining solution (Staining Solution: PI Solution: RNase A Solution = 500:10:10) was added to each cell pellet, and the mixture was gently pipetted and mixed. The cells were then wrapped in aluminum foil to protect them from light and incubated at 37 °C for 30 minutes for staining. The stained cells were analyzed using a flow cytometer (BECKMAN, Brea, California, USA), and the data were processed using FlowJo software.

### 2.14 Protein blotting

Colon cancer cells were lysed in a protein extraction reagent containing protease inhibitors. Protein concentrations in the samples were normalized using a BCA concentration kit. Proteins were separated by SDS-PAGE electrophoresis, transferred to PVDF membranes, and incubated with 5% skim milk powder for 2h. The membranes were then incubated with primary antibody at 4 °C for 24 h, followed by incubation with secondary antibody at 25 °C for 2h. PVDF membranes were exposed using a chemiluminescence imaging system (Jena, Germany), and the grayscale of each gel was calculated using ImageJ software.

### 2.15 Statistical analysis

All experiments were performed in three independent biological replicates. Data are presented as mean ± standard deviation (SD). Differences among multiple groups were analyzed using one-way analysis of variance (ANOVA) followed by Tukey’s *post hoc* test. A p value < 0.05 was considered statistically significant. Statistical analyses and graph generation were performed using GraphPad Prism 9.5.

## 3 Results

### 3.1 Targets of colon cancer and Typhonii Rhizoma

Using the keyword “colon cancer” to identify disease-related targets, 773 colon cancer-related targets were identified and deduplicated from GeneCards and OMIM. Subsequently, 466 TR-related targets were predicted from BATMAN-TCM and HERB. After standardization, 101 cross-targets between colon cancer-related targets and TR-related targets were identified as potential TR targets against colon cancer (**Figure 1A**).

### 3.2 Screening of drug-disease co-targets and construction of PPI network

The 101 overlapping targets were imported into the STRING database for protein-protein interaction (PPI) analysis. STRING was used to retrieve known and predicted protein interaction relationships and to explore the interaction characteristics among the common targets. The analysis was performed with the organism restricted to Homo sapiens, and the minimum required interaction score was set to 0.400. The resulting PPI network was visualized in Cytoscape (**Figures 1B, C**). Hub genes were further identified using the CytoHubba plugin, and the top-ranked genes, including AKT1, CASP3, EGFR, JUN, MAPK1, MAPK3, MYC, SRC, TP53, and VEGFA, are shown in **Figure 1D** and listed in **Table 2**.

**Table 2:** The hub genes were screened from the PPI network by CytoHubba.

Gene symbol	Stress	Degree	Neighborhood connectivity	Number of undirecred edges	Radiality	Betweenness
TP53	4744	91	46.417	91	0.99901	0.0369488
AKT1	4286	89	46.966	89	0.99879	0.0312809
EGFR	3562	83	47.843	83	0.99813	0.0282683
MYC	3166	82	49.621	82	0.99802	0.0173832
MAPK3	3120	81	49.037	81	0.99791	0.0202597
SRC	3672	80	48.125	80	0.99780	0.0405559
JUN	2950	79	50.354	79	0.99769	0.0163157
VEGFA	2694	78	50.474	78	0.99758	0.0189530
MAPK1	2632	77	50.844	77	0.99747	0.0152713
CASP3	2678	77	50.610	77	0.99747	0.0150309

### 3.3 Enrichment analysis of GO and KEGG

**Figure 2:**
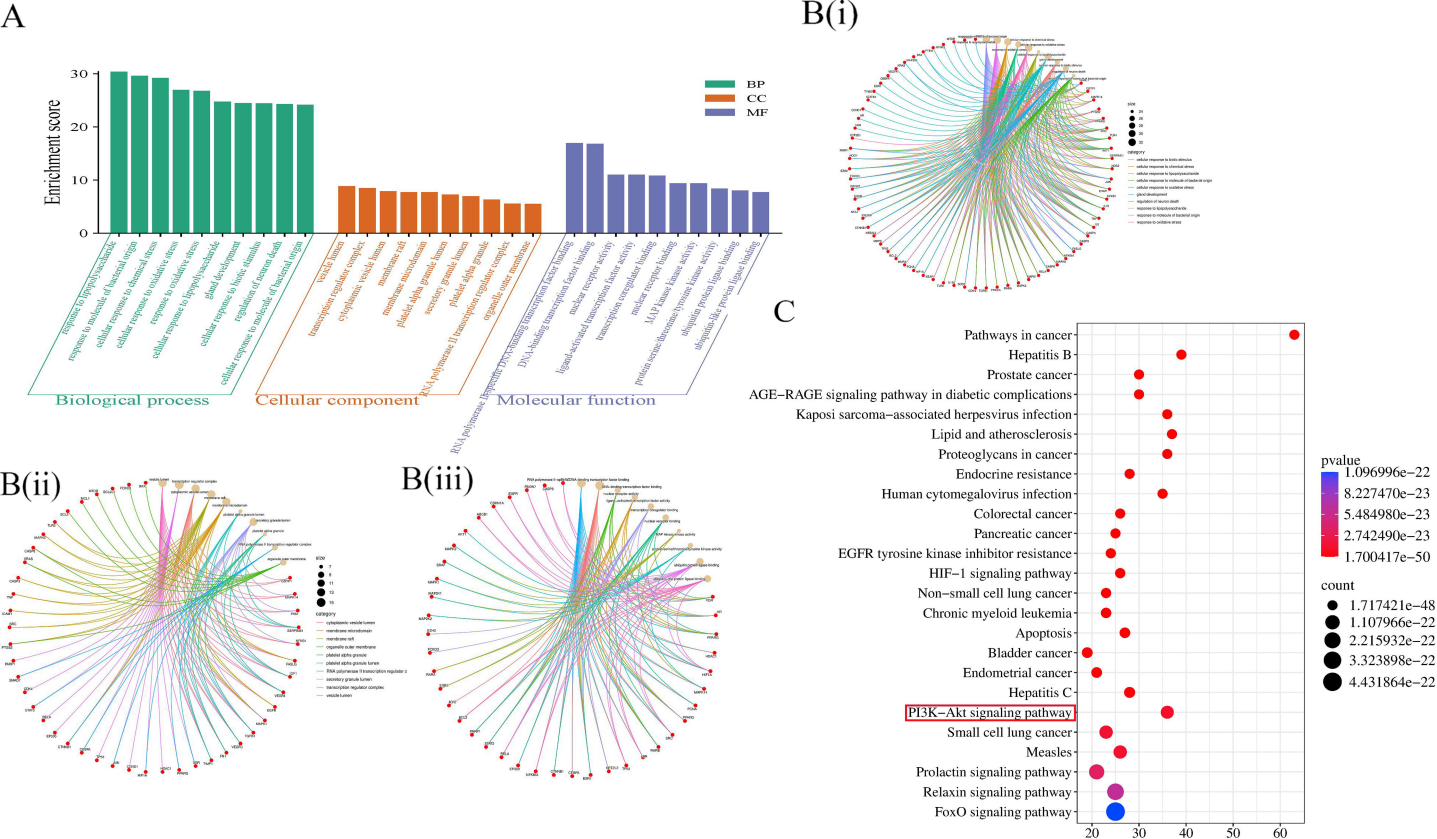
GO and KEGG enrichment analysis at the intersection of Typhonii Rhizoma and colon cancer target. **(A, B)** BP, CC, and MF enrichment analysis; Y-axis represents the ratio of enriched target genes/background genes. **(C)** Bubble diagram of the KEGG pathway.

**Figure 3:**
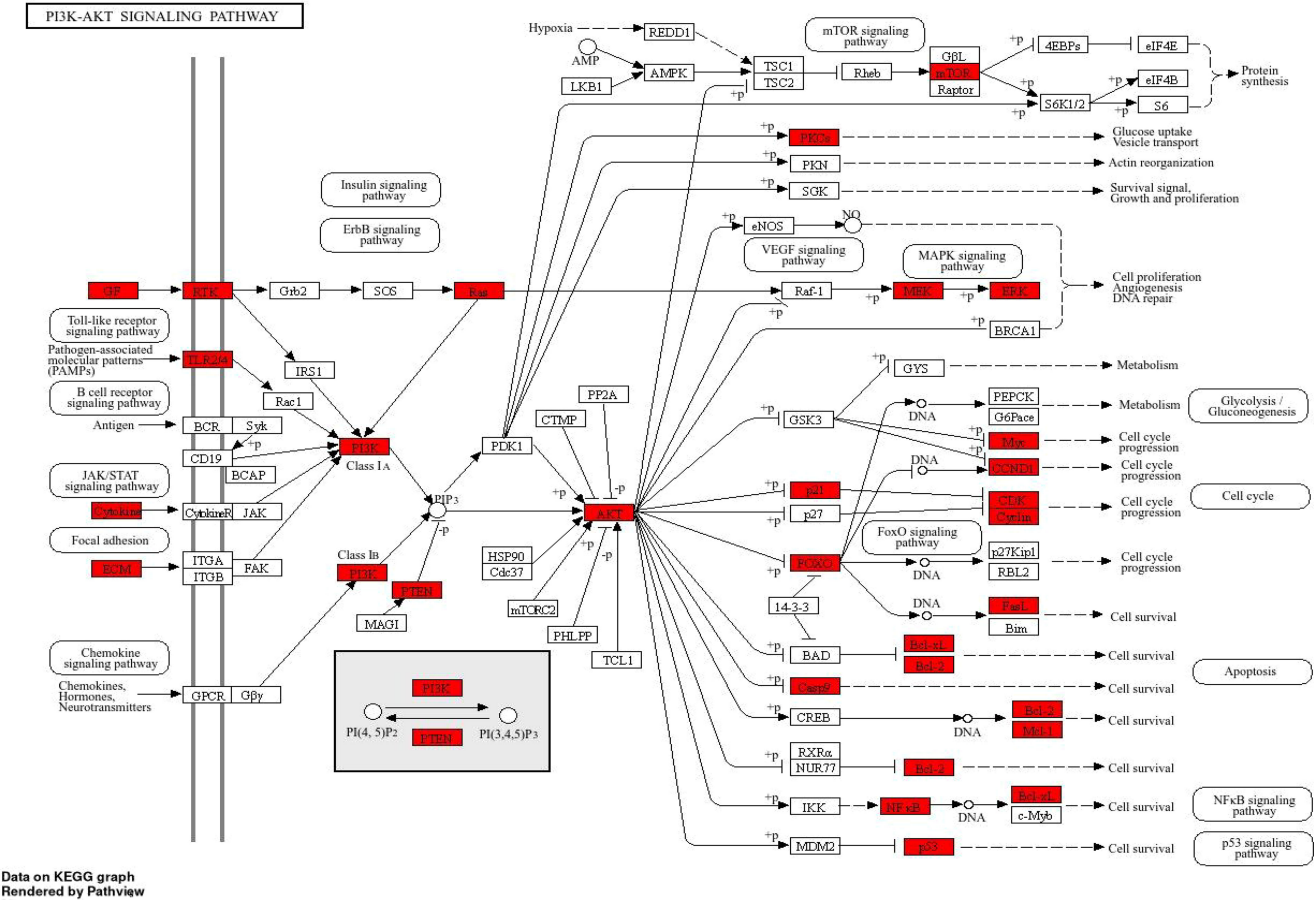
PI3K-AKT signaling pathway (red marks represent potential targets of white aconite intervention).

The DAVID database (33) is a relational database management system. Its core functions include: importing cross-targets from Gene Ontology (GO) and Kyoto Encyclopedia of Genes and Genomes (KEGG) into the database for enrichment analysis to identify potential treatment options and targets; and supporting the use of a visualization platform (https://davidbioinformatics.nih.gov/) to display GO terms and KEGG pathways. 101 potential colon cancer treatment targets of Typhonii Rhizoma were imported into DAVID for GO and KEGG enrichment analysis. The GO enrichment analysis selected the top 10 entries of each analysis type, BP (biological process), CC (cellular components), and MF (molecular function) for visualization in bioinformatics (**Figures 2A, B**). The 101 targets are mainly involved in biological processes such as response to lipopolysaccharide, response to molecules of bacterial origin, cellular response to chemical stress, and cellular response to oxidative stress. Lipopolysaccharide is a major surface component of Gram-negative bacteria and an important trigger of inflammatory responses (40). In colon cancer, gut microbiota dysbiosis and bacterial translocation have been reported to be associated with carcinogenesis (41). These enrichment results suggest that some predicted targets of TR may be associated with inflammation-related signaling. However, because the present study did not directly evaluate microbiota- or inflammation-related biomarkers, no mechanistic conclusion should be drawn from enrichment analysis alone. It mainly acts on vesicle lumen, cytoplasmic vesicle lumen, secretory granule lumen, platelet alpha granule, etc., which are all related to vesicle transport and secretion. In cancer cells, vesicle transport is abnormally active and is used to secrete growth factors, matrix metalloproteinases (promoting invasion) (42, 43), and exosomes (for intercellular communication and immune escape) (44). The main molecular functions involved include RNA polymerase II-specific DNA-binding transcription factor binding, DNA-binding transcription factor binding, nuclear receptor activity, ligand-activated transcription factor activity, etc., indicating that TR may bind to these transcription factors to directly activate or inhibit the activity of these transcription factors, thereby changing the expression profile of downstream genes on a large scale, regulating many genes related to cell proliferation, apoptosis, differentiation, and inflammation from the “source”, and achieving a synergistic effect of multiple targets and multiple pathways. KEGG pathway enrichment analysis screened 259 signaling pathways and visualized the first 25 pathways(**Figure 2C**), mainly involving Pathways in cancer, Proteoglycans in cancer, Colon cancer, EGFR tyrosine kinase inhibitor resistance, HIF-1 signaling pathway, PI3K-AKT signaling pathway, etc. Constitutive activation of the PI3K-AKT signaling pathway is a marker of colon tumor growth (45). As an important pathway, this pathway was selected for mapping (**Figure 3**). The red marks in the figure represent potential targets of white aconite intervention.

### 3.4 Construction of the disease-pathway-target-component-drug network

Based on the active components of Typhonii Rhizoma, the overlapping targets, and the enriched KEGG pathways, a disease-pathway-target-component-drug network was constructed in Cytoscape (**Figure 1E**). This network visually illustrates the relationships among Typhonii Rhizoma, its candidate active components, potential therapeutic targets, key signaling pathways, and colon cancer, reflecting the multi-component, multi-target, and multi-pathway characteristics of TR in colon cancer intervention.

### 3.5 Screening of core targets

In Cytoscape 3.10.3’s “cytohubba”, the top 10 targets are selected using degree, maximum neighborhood component (MNC), maximum clan centrality (MCC), and closeness. The intersection of the targets obtained by these four calculation methods is the core target.

### 3.6 Expression analysis of pivot genes

#### 3.6.1 mRNA expression level of core targets

**Figure 4:**
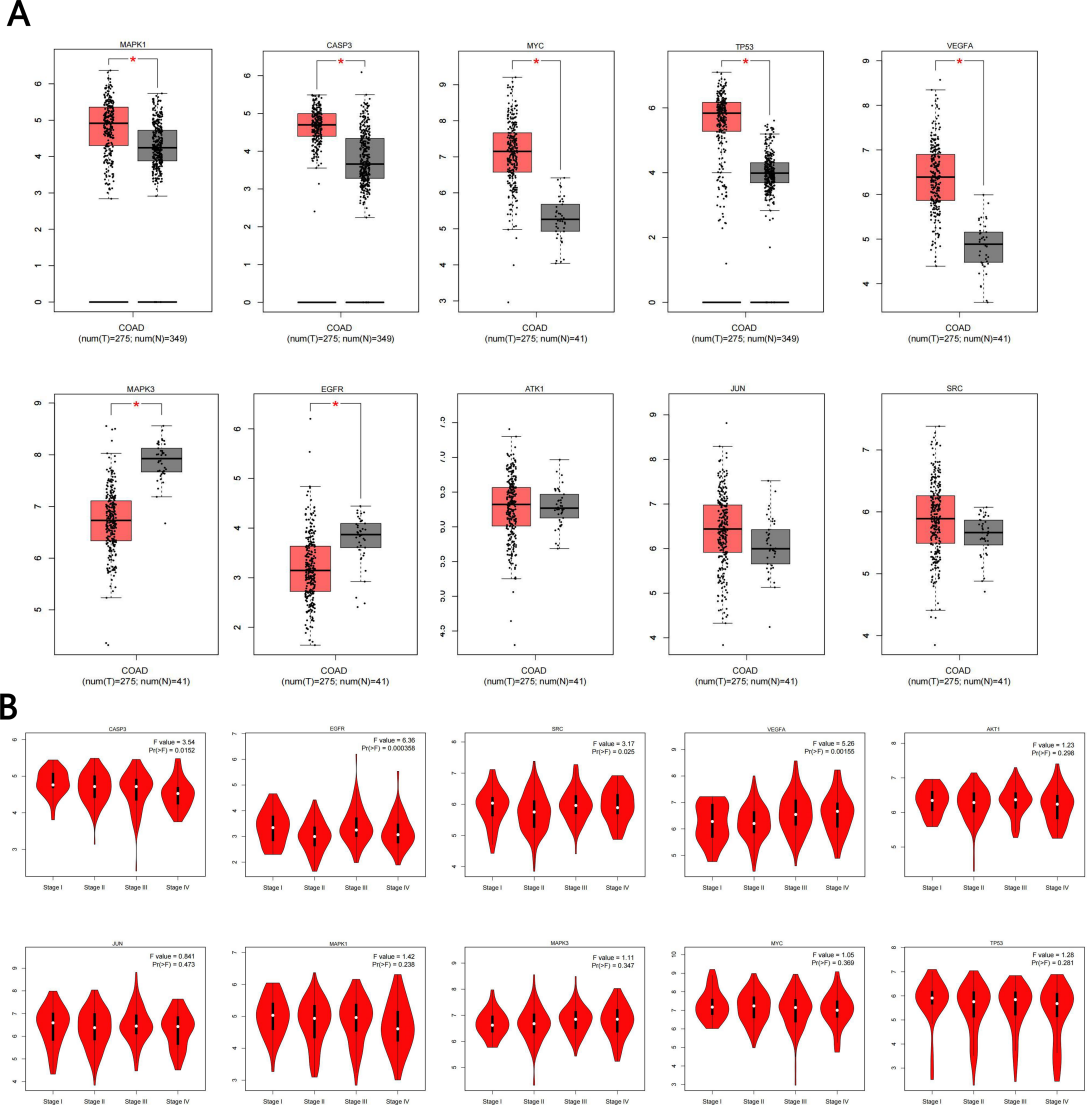
Hub gene expression in the GEPIA database. **(A)** Box plot of hub gene mRNA expression levels in the GEPIA database. Red represents tumor tissues and gray represents normal tissues. **(B)** Stage diagram of hub gene mRNA expression levels and pathological stages in the GEPIA database.

The expression of core targets differed between colon cancer and normal tissues. The mRNA levels of CASP3, MYC, MAPK1, TP53, and VEGFA were significantly higher in colon cancer than in normal tissues (*p* < 0.05), while the mRNA levels of MAPK3 and EGFR were significantly lower in colon cancer than in normal tissues (*p* < 0.05) (**Figure 4A**). Furthermore, we analyzed the relationship between the mRNA levels of core targets and the pathological stage of colon cancer. The results showed that the mRNA levels of CASP3, EGFR, SRC, and VEGFA changed significantly with pathological stage (*p* < 0.05) (**Figure 4B**).

#### 3.6.2 Protein expression levels of core targets

**Figure 5:**
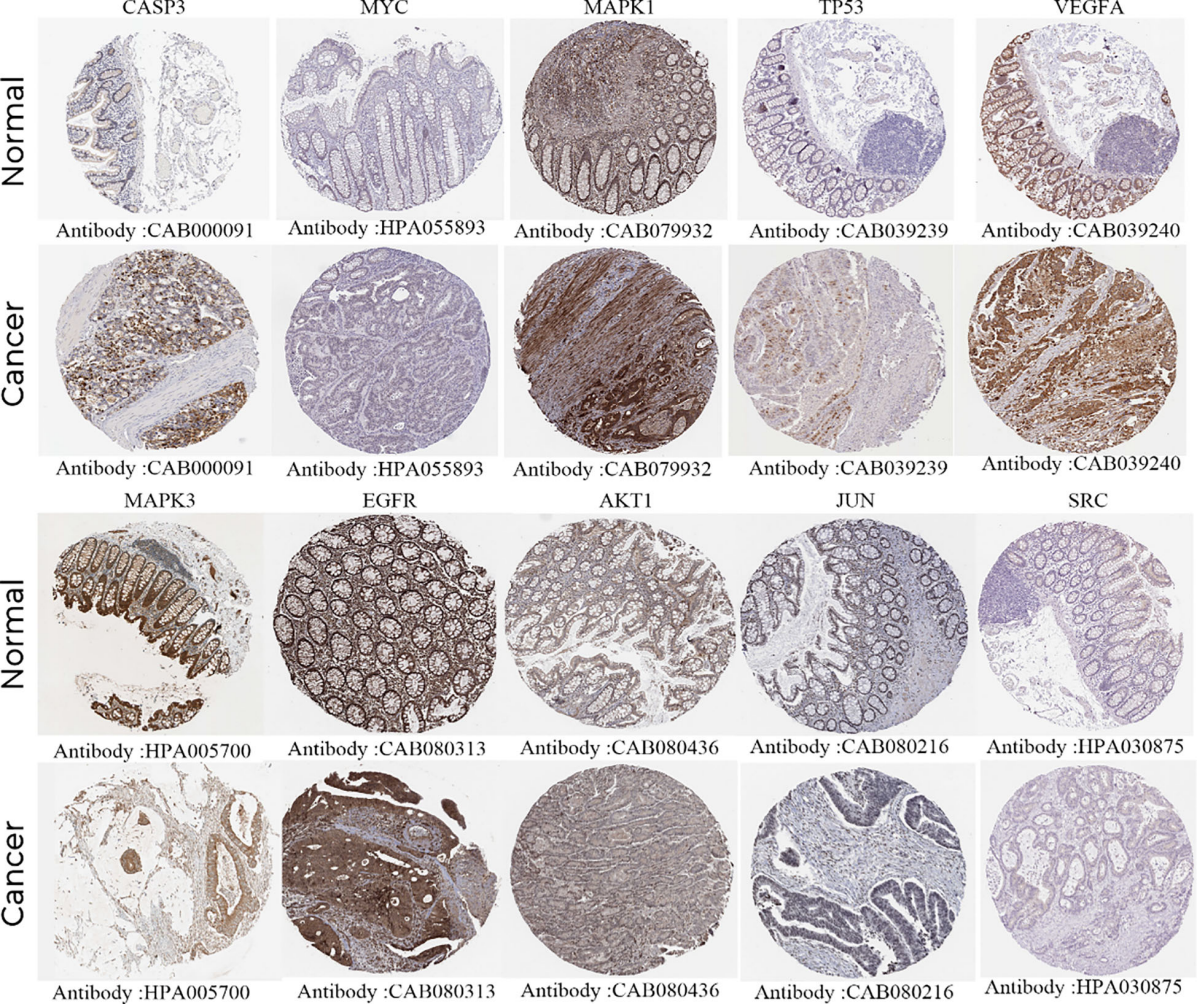
Immunohistochemical images of hub gene protein expression levels in the HPA database.

Immunohistochemical staining images from the HPA database were analyzed to observe the expression levels of core target proteins in colon cancer. We found that compared with normal colonic tissue, the expression levels of CASP3, MYC, MAPK1, TP53, AKT1, and VEGFA were increased in colon cancer tissue, while the expression levels of MAPK3 and EGFR were decreased, and the expression levels of JUN and SRC were basically the same (**Figure 5**).

#### 3.6.3 Prognostic relevance of hub genes

To further reduce target-selection bias and evaluate the potential clinical relevance of the identified hub genes, prognostic analysis was performed for the top 10 hub genes using publicly available colon cancer datasets in Sangerbox 3.0. The results showed that the hub genes exhibited heterogeneous associations with overall survival, indicating that high topological ranking did not necessarily correspond to significant prognostic relevance. Notably, AKT1 was not significantly associated with overall survival in the analyzed cohort (p = 0.17). Therefore, AKT1 was not prioritized because of prognostic dominance. Instead, it was retained as a candidate hub target primarily because of its high ranking in the PPI network, its central position within the PI3K/AKT signaling pathway, supportive protein-level expression evidence, and the observed reduction in p-AKT/AKT signaling following TR treatment *in vitro*. Taken together, these results suggest that AKT1 should be interpreted as a mechanistically prioritized candidate hub target rather than a survival-supported dominant target. The prognostic results of the top 10 hub genes are summarized in **Table 3**, and the corresponding Kaplan–Meier survival curves are provided in **Supplementary Figure 1**.

**Table 3:** Prognostic analysis of the top 10 hub genes in the TCGA-COAD cohort.

Gene	Cohort	Endpoint	Cutoff	HR	95% CI	Log-rank P value	Association with OS
TP53	TCGA-COAD via Sangerbox 3.0	OS	Median	0.54	0.32-0.89	0.01	significant
AKT1	TCGA-COAD via Sangerbox 3.0	OS	Median	0.68	0.39-1.18	0.17	Not significant
EGFR	TCGA-COAD via Sangerbox 3.0	OS	Median	0.72	0.45-1.16	0.18	Not significant
MYC	TCGA-COAD via Sangerbox 3.0	OS	Median	0.76	0.47-1.24	0.27	Not significant
MAPK3	TCGA-COAD via Sangerbox 3.0	OS	Median	1.61	0.98-2.67	0.06	Not significant
SRC	TCGA-COAD via Sangerbox 3.0	OS	Median	1.54	0.91-2.61	0.11	Not significant
JUN	TCGA-COAD via Sangerbox 3.0	OS	Median	0.65	0.36-1.19	0.16	Not significant
VEGFA	TCGA-COAD via Sangerbox 3.0	OS	Median	1.85	1.14-3.00	0.01	significant
MAPK1	TCGA-COAD via Sangerbox 3.0	OS	Median	0.48	0.30-0.79	2.80E-03	significant
CASP3	TCGA-COAD via Sangerbox 3.0	OS	Median	0.57	0.35-0.92	0.02	significant

### 3.7 Molecular docking

**Figure 6:**
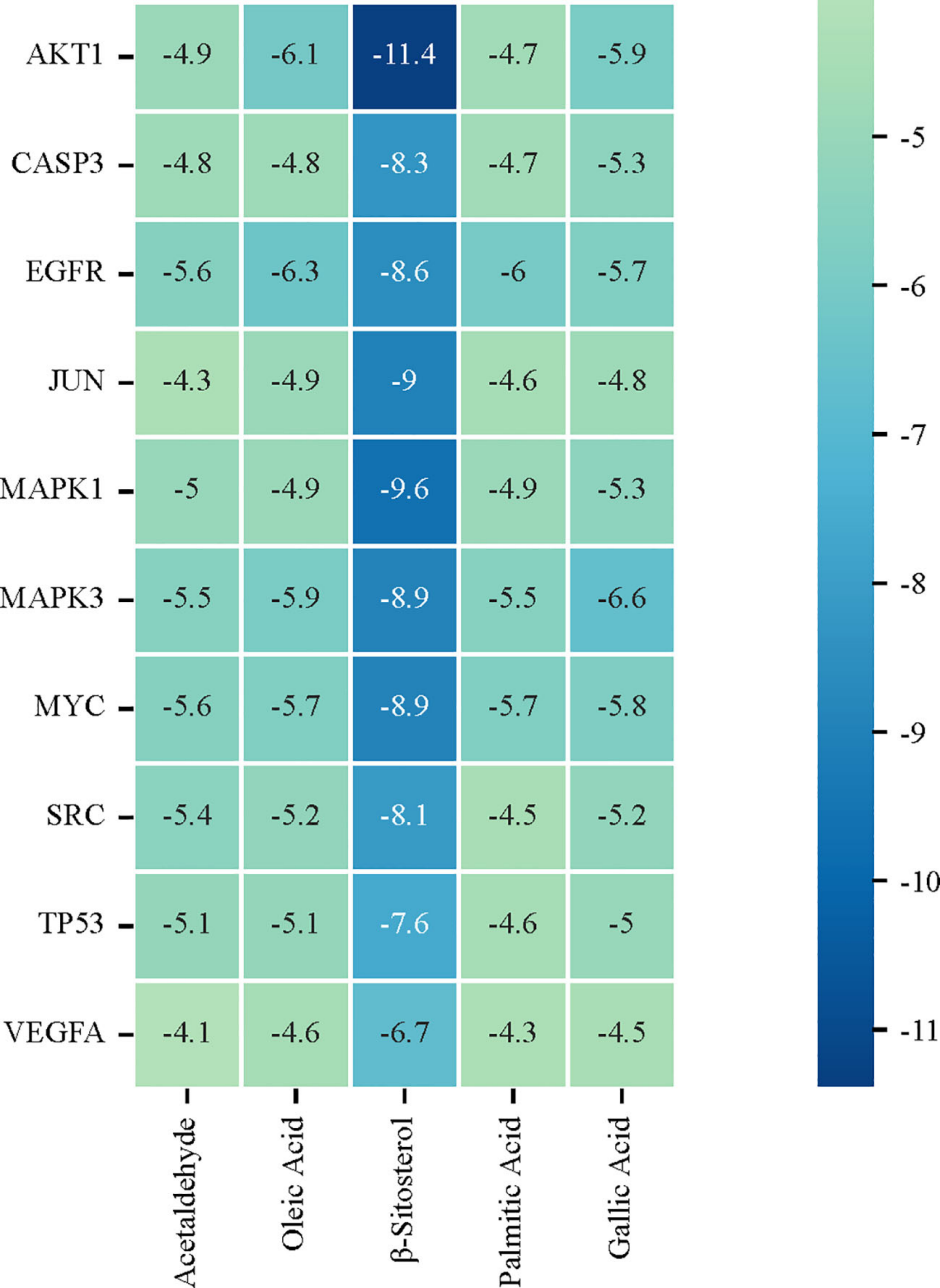
Molecular docking results between candidate core active ingredients and core genes.

**Figure 7:**
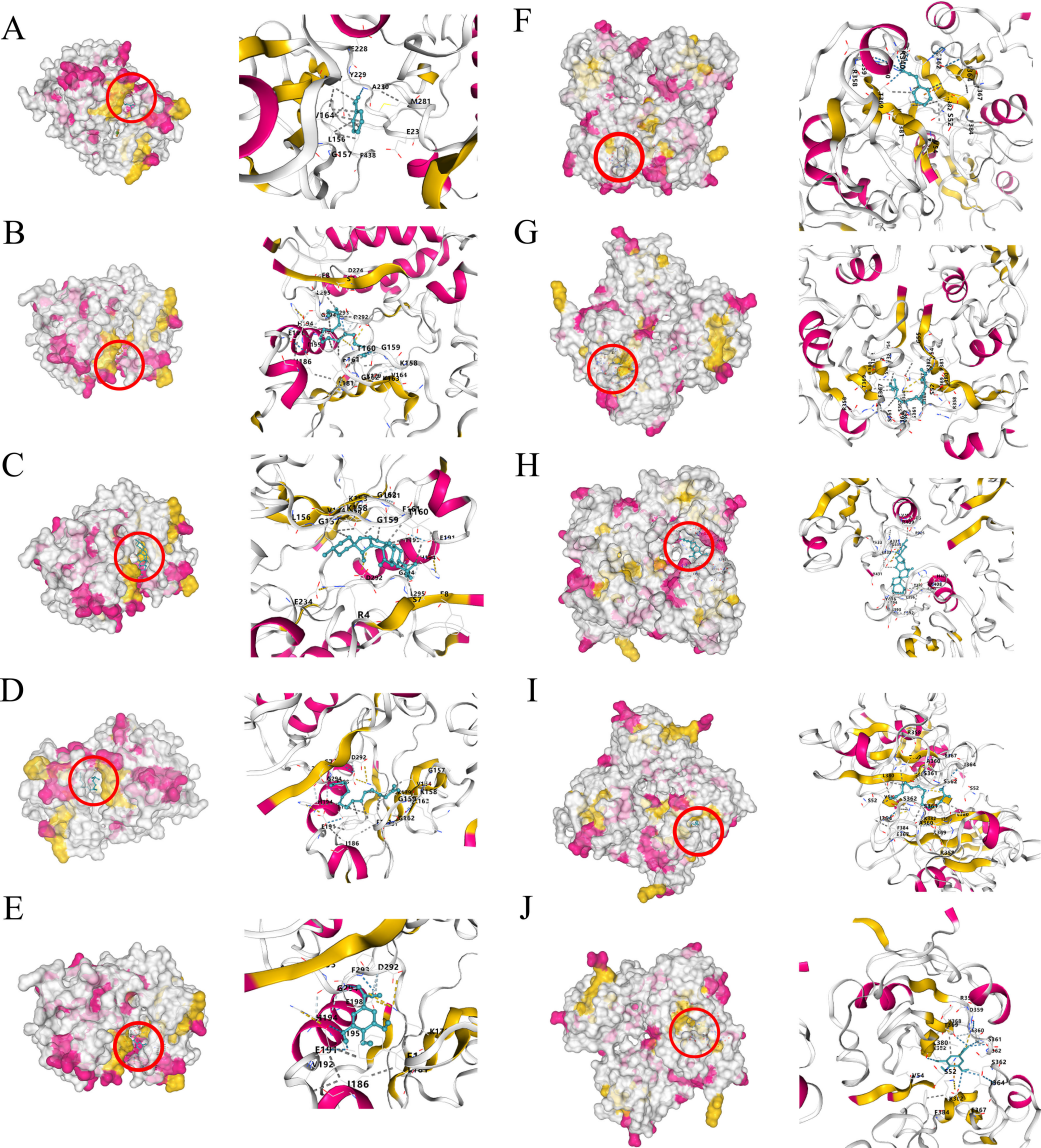
Molecular docking results of important compounds and proteins corresponding to key gene targets in related signaling pathways. **(A)** Acetaldehyde - AKT1, **(B)** Oleic Acid - AKT1, **(C)** Palmitic Acid - AKT1, **(D)** β-Sitosterol - AKT1, **(E)** Gallic Acid - AKT1, **(F)** Acetaldehyde - PI3K, **(G)** Oleic Acid - PI3K, **(H)** Palmitic Acid - PI3K, **(I)** β-Sitosterol - PI3K, **(J)** Gallic Acid - PI3K.

The PPI network diagram of potential targets was analyzed using CytoHubba (**Figure 1C**), and 10 core targets were selected (**Figure 1D**). Five key components of Typhonii Rhizoma were screened based on their degree values using CytoNCA. Molecular docking results showed that the binding energies of these five key components to the target proteins were all less than 0 (**Figure 6**). These findings suggest that several candidate components of Typhonii Rhizoma may have favorable interaction tendencies with the selected core target proteins, especially AKT1 and PI3K. However, because molecular docking is a computational prediction, the docking scores alone cannot be regarded as direct evidence of target inhibition or strong binding. Therefore, the docking results in this study should be considered supportive evidence for the predicted component-target associations. KEGG analysis suggested that TR may exert its effects through the PI3K/AKT signaling pathway in colon cancer. Among the hub genes identified by network topology analysis, AKT1 was prioritized as a candidate hub target because it ranked highly in the PPI network and occupied a central position in the PI3K/AKT signaling axis. Although AKT1 did not show a significant difference at the mRNA level in the GEPIA database, HPA data suggested increased protein-level expression in colon cancer tissues. This inconsistency between mRNA and protein expression may reflect post-transcriptional or post-translational regulation. Therefore, the current evidence supports the prioritization of AKT1 as a candidate hub target for subsequent validation, rather than definitive confirmation of direct target involvement. Accordingly, AKT1 (PDB: 3QKL) and PI3K (PDB: 7RNU) were selected as representative targets for molecular docking. Theoretically, ligand-receptor complexes with lower binding energies are considered to have more stable conformations (46). Taken together, these docking results suggest that several candidate components of TR may interact favorably with AKT1 and PI3K at the structural level (**Figure 7**).

### 3.8 Experimental verification

#### 3.8.1 TR inhibits the activity of colon cancer cells

**Figure 8:**
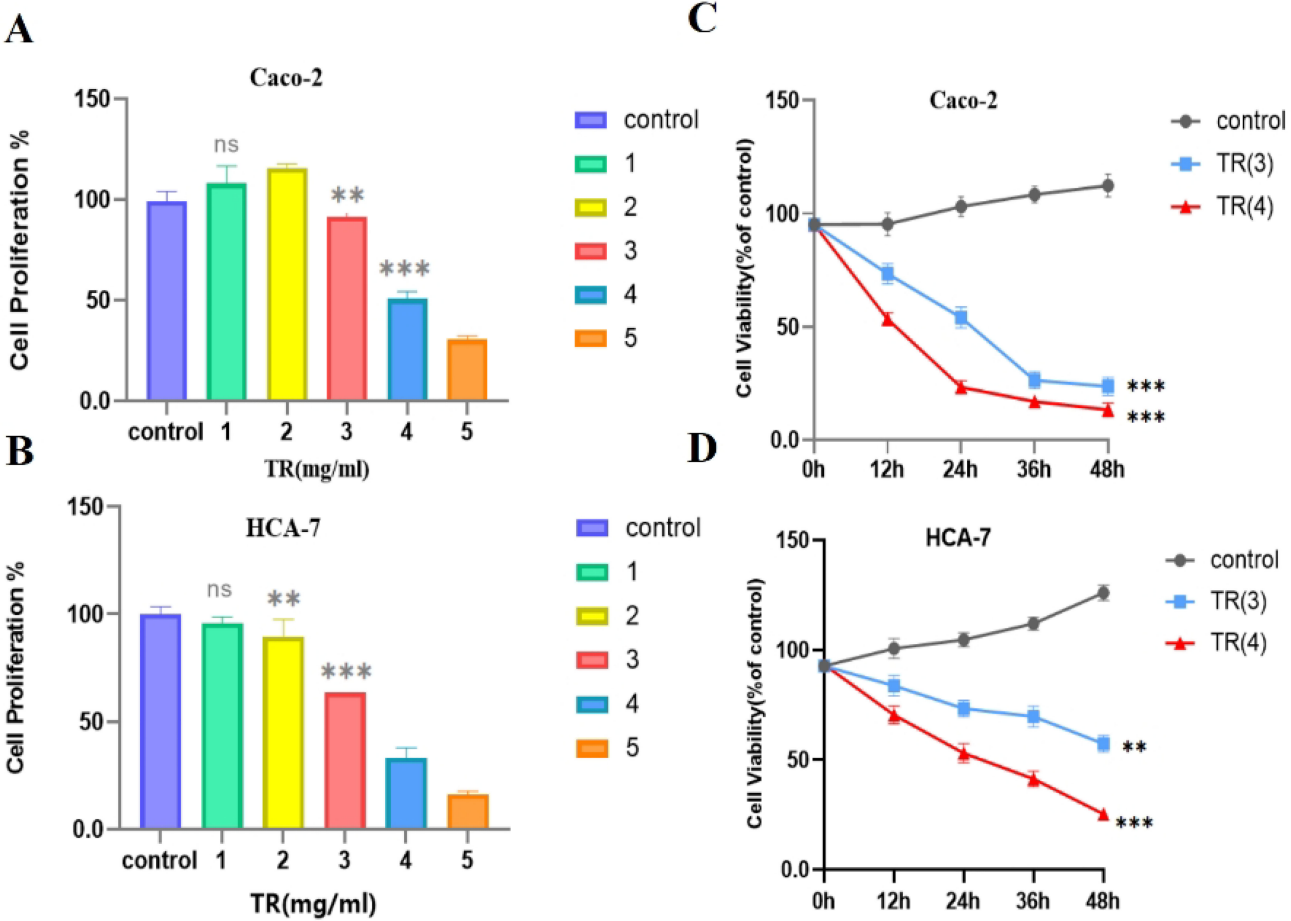
Effects of TR on colon cancer cell proliferation. **(A)** Effects of different concentrations of TR on Caco-2 cell proliferation. **(B)** Effects of different concentrations of TR on HCA-7 cell proliferation. **(C)** Effects of TR concentration at different time points on Caco-2 cell proliferation. **(D)** Effects of TR concentration at different time points on HCA-7 cell proliferation. (Compared with the vehicle control group, ns P > 0.05, *P < 0.05, **P < 0.01, ***P < 0.01).

Based on network pharmacology and molecular docking results, we further explored and validated the potential therapeutic effect of TR on colon cancer through *in vitro* experiments. To assess the effect of TR on colon cancer cells, we analyzed the cell viability of Caco-2 and HCA-7 cells treated with different doses of TR. All *in vitro* comparisons were made against the vehicle control group, and no pathway-selective pharmacologic comparator was included in the current experimental design. The results showed that TR treatment significantly inhibited the cell viability of both Caco-2 and HCA-7 cells. Specifically, the cell viability of Caco-2 cells decreased after 24 h of treatment with TR concentrations ranging from 3mg/mL to 5mg/mL, while the cell viability of HCA-7 cells decreased after 24h of treatment with TR concentrations ranging from 1mg/mL to 5mg/mL (**Figures 8A, B**). We then used TR concentrations of 3mg/mL and 4mg/mL to detect the viability of Caco-2 and HCA-7 cells treated with this traditional Chinese medicine at different time points (0h, 12h, 24h, 36h, and 48h). The results indicated that the viability of both cell types decreased with prolonged drug treatment time (**Figures 8C, D**). These findings suggest that TR inhibits the viability of colon cancer cells in a concentration- and time-dependent manner.

#### 3.8.2 TR inhibits the wound closure and invasion ability of colon cancer cells

**Figure 9:**
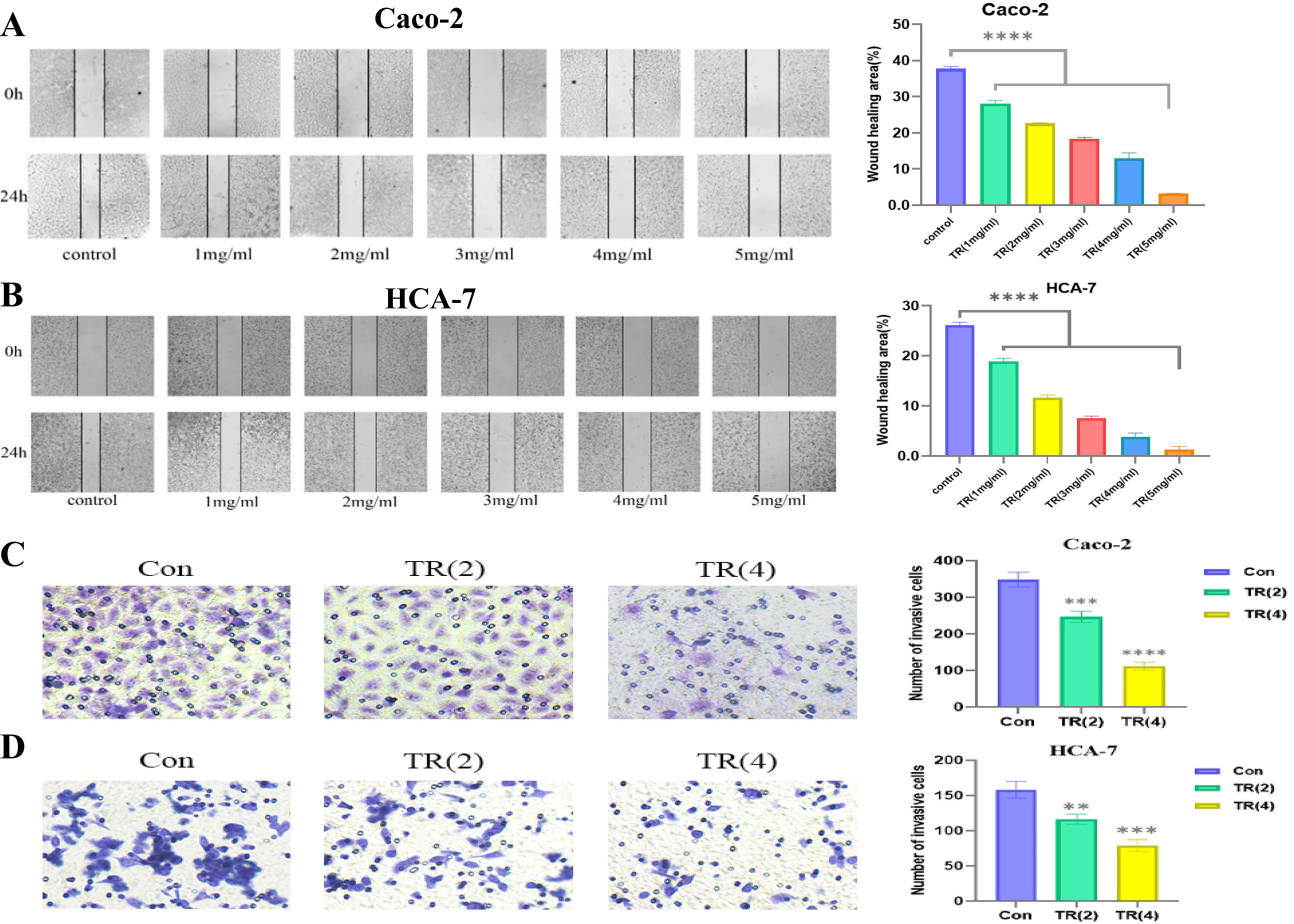
Effects ofTR on the migration and invasion of colon cancer cells. **(A)** Effect of TR on the migration of Caco-2 cells after 24 h of treatment and statistical analysis. **(B)** Effect of TR on the migration of HCA-7 cells after 24 h of treatment and statistical analysis. **(C)** Effect of TR on the invasion of Caco-2 cells after 24 h of treatment and statistical analysis. **(D)** Effect of TR on the invasion of HCA-7 cells after 24 h of treatment and statistical analysis. (Compared with the vehicle control group, *P<0.05, **P<0.01, ***P<0.001, ****P<0.0001).

To investigate the effects of TR on the migration and invasion of colon cancer cells, we performed wound healing and Transwell invasion assays to explore the potential inhibitory effect of TR on colon cancer migration and invasion. **Figures 9A, B** show that untreated Caco-2 and HCA-7 cells exhibited strong migration ability, while TR treatment significantly and concentration-dependently inhibited the migration of these cell lines. Furthermore, **Figures 9C, D** show that untreated Caco-2 and HCA-7 cells exhibited strong invasive ability, while the number of transmembrane cells was significantly reduced in the TR-treated group, and the invasion of these cell lines was inhibited in a concentration-dependent manner. In summary, these findings indicate that TR can significantly inhibit the migration and invasion of colon cancer cells in a concentration-dependent manner.

#### 3.8.3 Effect of TR on apoptosis in colon cancer cells

**Figure 10:**
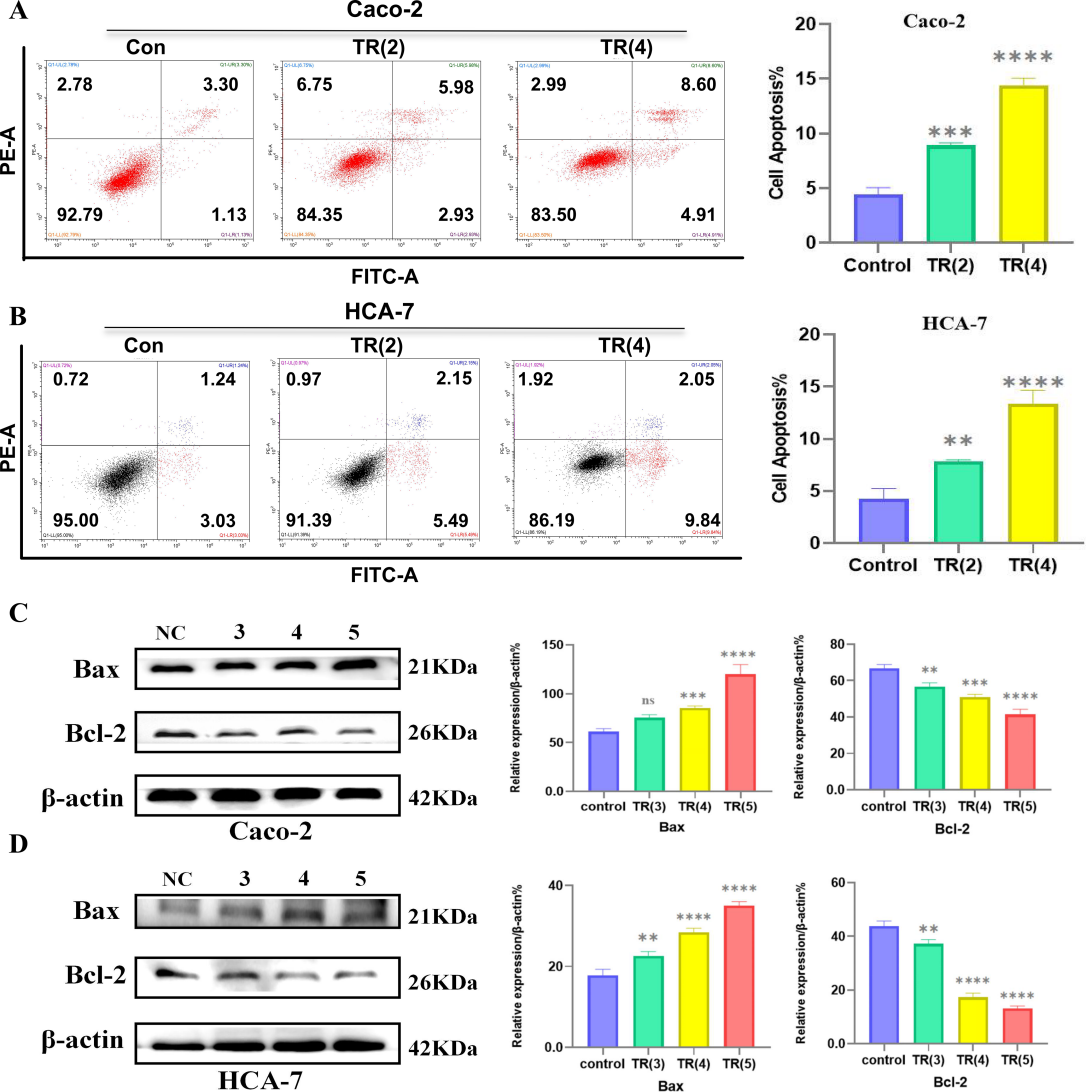
TR-induced mitochondrial-dependent apoptosis. **(A)** Flow cytometry analysis of apoptosis in Caco-2 cells and statistical analysis. **(B)** Flow cytometry analysis of apoptosis in HCA-7 cells and statistical analysis. **(C)** Western blot analysis of Bcl-2 and Bax in Caco-2 cells. **(D)** Western blot analysis of Bcl-2 and Bax in HCA-7 cells. (ns P > 0.05, *P < 0.05, **P < 0.01, ***P < 0.001, ****P < 0.0001).

TR, due to its anti-colon cancer effect, is also enriched in the apoptotic pathway. Based on this, we attempted to investigate whether TR could induce apoptosis in colon cancer cells. **Figures 10A, B** show that TR treatment significantly increased apoptosis in both types of colon cancer cells, with a significant increase in the proportion of late-stage apoptotic and necrotic cells. Furthermore, Western blot analysis revealed that TR treatment inhibited Bcl-2 expression while increasing Bax expression, leading to a sharp decrease in the Bcl-2/Bax ratio. These results indicate that TR drives apoptosis by regulating the mitochondrial-mediated endogenous apoptotic pathway.

#### 3.8.4 TR arrests the cell cycle in G0/G1 phases

**Figure 11:**
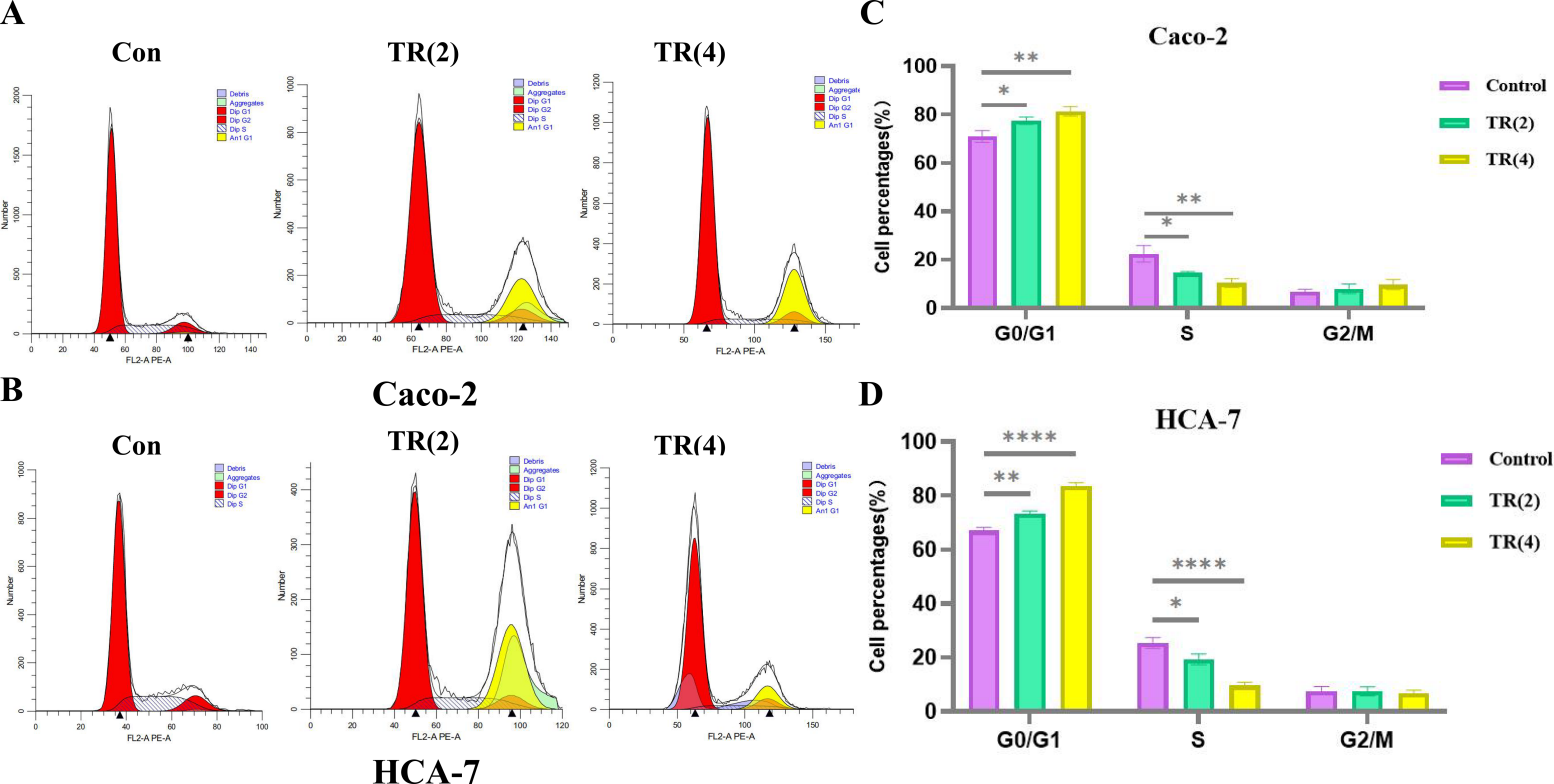
Effects ofTR on the cell cycle of colon cancer cells. **(A, B)** Cell cycle changes and statistical analysis of Caco-2 cells after 24 h of treatment with different concentrations of TR. **(C, D)** Cell cycle changes and statistical analysis of HCA-7 cells after 24 h of treatment with different concentrations of TR. (Compared with the vehicle control group, ns P > 0.05, *P < 0.05, **P < 0.01, ***P < 0.001, ****P < 0.0001).

In Caco-2 and HCA-7 cells, as TR concentration increased, the number of cells in G1 phase increased significantly, while the number of cells in S phases decreased, indicating that TR can induce G0/G1 phase arrest (P < 0.01), thereby inhibiting cell proliferation (**Figure 11**).

#### 3.8.5 TR inhibits the PI3K/AKT signaling pathway in colon cancer cells

**Figure 12:**
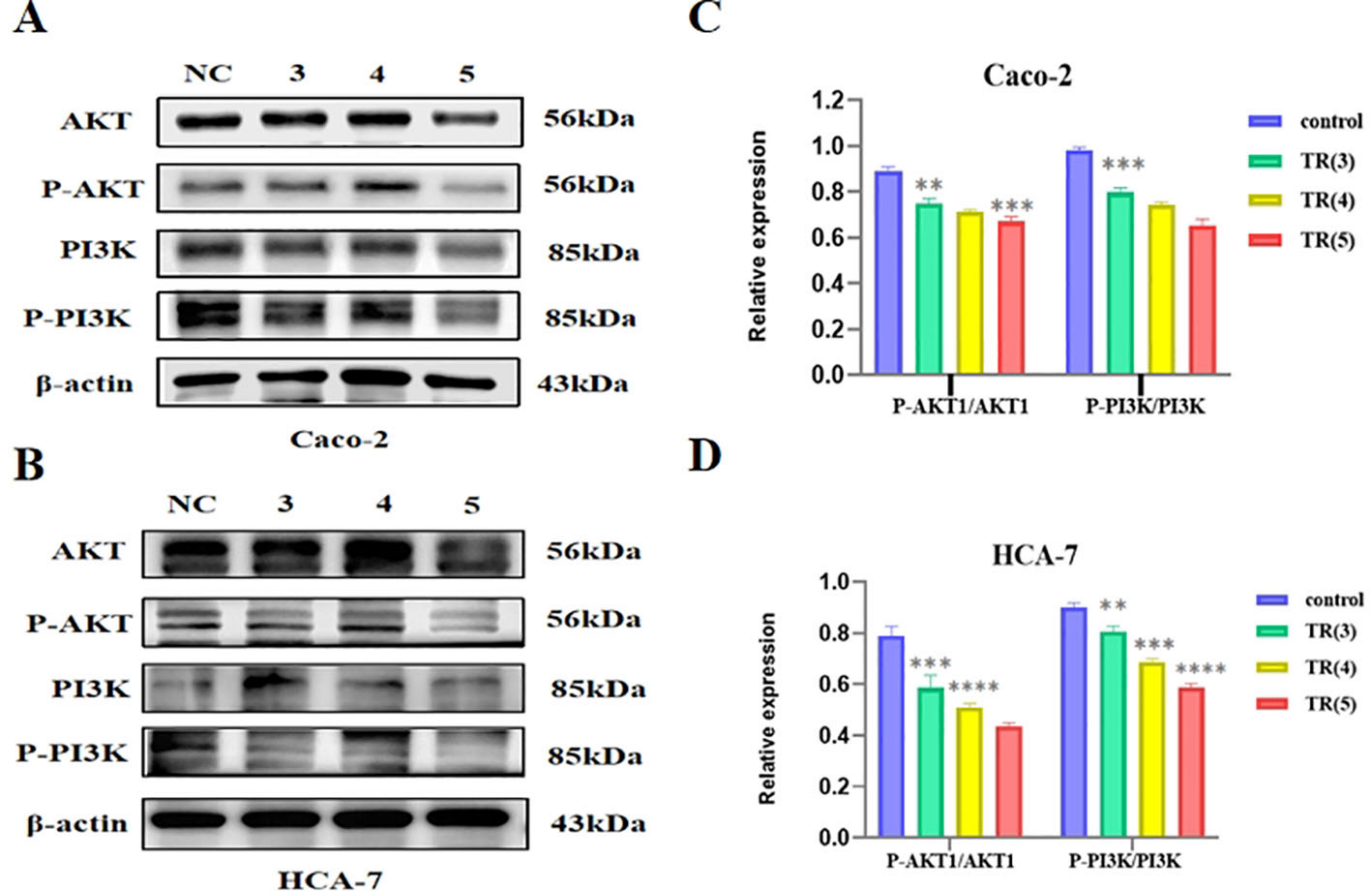
Western blot analysis of PI3K/AKT signaling pathway expression by TR. **(A)** Analysis of total and phosphorylated proteins related to the PI3K/AKT signaling pathway by TR in Caco-2 cells. **(B)** Analysis of total and phosphorylated proteins related to the PI3K/AKT signaling pathway by TR in HCA-7 cells. **(C)** Statistical bar chart of the ratio of phosphorylated protein to total protein grayscale value in Caco-2 cells. **(D)** Statistical bar chart of the ratio of phosphorylated protein to total protein grayscale value in HCA-7 cells.(Compared with the vehicle control group, ns P > 0.05, *P < 0.05, **P < 0.01, ***P < 0.001, ****P < 0.0001).

These *in vitro* experimental results indicate that TR inhibits cell viability, invasion capability, and wound closure in colon cancer cells. Furthermore, KEGG, GO, and molecular docking analyses suggest that TR may regulate the PI3K/AKT signaling pathway in colon cancer. To further explore the mechanism of action of TR in colon cancer, we investigated the effect of TR on the PI3K/AKT signaling pathway in colon cancer cells. The results showed that TR significantly reduced the protein expression ratios of p-PI3K/PI3K and p-AKT/AKT in colon cancer cells. These findings are consistent with the possibility that TR suppresses colon cancer cell growth in association with reduced PI3K/AKT pathway activity (**Figure 12**).

## 4 Discussion

Colon cancer (CC), as a highly heterogeneous malignant tumor, poses a significant challenge to clinical treatment due to its complex pathophysiological mechanisms and severe drug resistance (47–49). Therefore, the discovery of antitumor drugs with novel mechanisms of action and multi-target characteristics from natural products has become an important frontier in oncology research. Typhonii Rhizoma (TR), the dried tuber of Typhonium giganteum Engl. (Araceae family), possesses significant anti-inflammatory and anticancer activities (45, 50), but its molecular mechanism in colon cancer remains largely unexplained. In this study, we employed a comprehensive approach combining network pharmacology, molecular docking, and experimental validation to systematically reveal the core mechanism by which TR exerts its anti-colon cancer activity by inhibiting the PI3K/AKT signaling pathway, providing preliminary mechanistic support for further experimental investigation.

The network pharmacology analysis in this study is the starting point for elucidating the complex pharmacological effects of TR. The 101 TR-Colon cancer intersection targets we identified demonstrate the synergistic effect of traditional Chinese medicine, reflecting its “multi-component, multi-target” nature. The 10 hub genes identified in the PPI network, such as AKT1, TP53, MYC, VEGFA, and EGFR, are all recognized tumor drivers or inhibitors and are critically involved in almost all “hallmarks of cancer, “ such as cell proliferation, apoptosis, angiogenesis, and signal transduction (51, 52). This finding strongly suggests that the anticancer effect of TR does not depend on a single target, but is achieved through extensive modulation of multiple key biological network nodes in the development of colon cancer. Among these hub genes, AKT1 was prioritized for further analysis because of its high topological ranking and its central position within the PI3K/AKT pathway; however, the present evidence supports its prioritization as a candidate hub rather than definitive confirmation as a direct target of TR.

KEGG and GO functional enrichment analysis further aggregated these discrete targets into functionally defined biological pathways, thus providing clear hypotheses for exploring the underlying mechanisms. The results showed that the “PI3K-AKT signaling pathway” occupied a central position among all enriched pathways. The PI3K/AKT signaling pathway plays a crucial role in regulating a variety of cellular functions, including metabolism, growth, proliferation, survival, transcription, and protein synthesis, and contributes significantly to tumor emergence and progression (53). Studies have shown that the PI3K/AKT pathway is closely related to apoptosis, proliferation, invasion, metastasis, and radiotherapy and chemotherapy resistance in colon cancer (54). Abnormal activation of the PI3K/AKT pathway is frequently observed in invasive cancers, especially mutations in the PIK3CA gene encoding the PI3K p110α catalytic subtype (55, 56). Such mutations are common in many malignancies and contribute to tumorigenesis, including that of colon cancer. It is also noteworthy that GO enrichment highlighted several inflammation-related terms, including”response to lipopolysaccharide.”These findings may suggest a possible association with inflammation-related signaling; however, no microbiota- or cytokine-related measurements were performed in the present study. Therefore, this possibility should be regarded as a hypothesis requiring dedicated validation rather than as direct evidence that TR acts through gut microbiota-mediated inflammatory regulation (57). To explore the molecular plausibility of the interaction between the active components of TR and its core targets, we then performed molecular docking. The results showed that five key active components in TR, especially β-sitosterol, exhibited favorable docking scores with both PI3K and AKT1, suggesting potential interactions at the structural level. However, docking scores alone do not establish direct target inhibition or binding potency. Therefore, these findings should be interpreted as supportive computational evidence for the predicted component-target associations rather than definitive proof that TR components act as direct inhibitors of the PI3K/AKT pathway. Because the docking workflow was not benchmarked against known ligands or re-docking metrics in the present study, these results should be interpreted as hypothesis-supporting rather than methodologically definitive structure-based validation. When considered together with the network pharmacology results and the *in vitro* Western blot findings showing reduced p-PI3K/PI3K and p-AKT/AKT ratios, the docking data further support the possible involvement of the PI3K/AKT signaling pathway in the anti-colon cancer effects of TR.

The core value of this study lies in the subsequent *in vitro* experiments that systematically validated the in silico predictions, forming the strongest chain of evidence. First, phenotypic experiments (CCK-8 and scratch healing) directly demonstrated the significant inhibitory effect of TR extract on the viability and migration ability of Caco-2 and HCA-7 cells in a concentration- and time-dependent manner, clarifying its anti-colon cancer biological effect. Furthermore, invasion experiments revealed that TR treatment significantly reduced the number of invasive cells and inhibited the invasive ability of both Caco-2 and HCA-7 cell lines in a concentration-dependent manner (as shown in **Figures 9C, D**). More importantly, the mechanistic Western blot analysis precisely revealed the molecular events: after TR treatment, the protein expression ratios of p-PI3K/PI3K and p-AKT/AKT significantly decreased. The observed phenotypic changes, including reduced viability, increased apoptosis, and G0/G1 arrest, are consistent with reduced PI3K/AKT pathway activity; however, they should not be interpreted as exclusive evidence that PI3K/AKT is the only or uniquely specific mechanism involved. Other potentially relevant pathways identified in the enrichment analysis may also contribute and warrant further investigation. Meanwhile, flow cytometry analysis (**Figures 10A, B**) showed that TR treatment significantly increased the apoptosis rate in both colon cancer cell lines, particularly the proportion of late-stage apoptotic and necrotic cells. At the molecular level, Western blotting results showed that TR inhibited the expression of the anti-apoptotic protein Bcl-2 while promoting the expression of the pro-apoptotic protein Bax, leading to a sharp decrease in the Bcl-2/Bax ratio. This indicates that TR induces colon cancer cell apoptosis by regulating the intrinsic (mitochondrial) apoptosis pathway (**Figures 10C, D**). Thus, this study constitutes a complete evidence chain from computational prediction (network targets and pathways) to molecular simulation (component-target binding), then to cell phenotype (inhibition of proliferation/migration/invasion and induction of apoptosis), and finally to molecular mechanisms (inhibition of pathway activity and activation of apoptosis pathways).

The significance of this study lies in providing new mechanistic insights into TR as a candidate drug for the treatment of colon cancer. Unlike single-molecule inhibitors targeting the PI3K/AKT pathway currently used in clinical practice (such as Alpelisib), which are prone to rapid resistance development due to bypass activation or feedback loops (58, 59), TR, as a multi-component mixture, exhibits a “broad-spectrum” inhibitory profile, acting simultaneously on pivotal targets such as AKT1, EGFR, SRC, and MAPK1. Given the multi-target characteristics of TR, its potential relevance to resistance-related signaling warrants further investigation. Notably, this study also found that TR can significantly induce G0/G1-phase arrest in Caco-2 and HCA-7 cells (P<0.01), and this cell-cycle regulatory effect is closely related to inhibition of the PI3K/AKT pathway. The PI3K/AKT pathway plays a key regulatory role in cell-cycle progression; decreased activity leads to activation of cell-cycle checkpoints, thereby arresting cells in theG0/G1 phases and ultimately inhibiting cell proliferation. This multi-target pattern not only blocks proliferation-related signaling through PI3K/AKT pathway inhibition but may also enhance anti-tumor activity by promoting intrinsic apoptosis and cell-cycle arrest, thereby providing a basis for future studies on resistance-related signaling and rational therapeutic combination strategies.

Despite the positive findings of this study, several limitations should be acknowledged. First, this study used crude TR extract, and its anticancer activity is likely the result of the combined effects of multiple constituents. Accordingly, the exposure concentration was expressed as mass concentration (mg/mL) rather than molar concentration, which limits direct potency comparisons with studies using chemically defined compounds. Further activity-guided fractionation, purification, and monomer-level validation are therefore needed. Second, this study was limited to *in vitro* cell models and could not recapitulate the complex *in vivo* tumor microenvironment, including tumor–stromal–immune interactions and ADME-related processes. Thus, the findings should be interpreted as *in vitro* evidence only, and no conclusion can yet be drawn regarding the *in vivo* antitumor efficacy, systemic safety, or pharmacokinetic behavior of TR. In addition, no pathway-selective pharmacologic comparator (such as a PI3K or AKT inhibitor) was included, preventing assessment of the relative specificity and benchmark efficacy of TR against canonical PI3K/AKT inhibition. Furthermore, because only two colon cancer cell lines were examined, the generalizability of the observed effects across tumors with different driver gene alterations (such as KRAS and BRAF) remains uncertain. Finally, network pharmacology relies on database completeness and accuracy, and in this study herb-related targets were retrieved directly from BATMAN-TCM and HERB without an initial OB/DL-based prefilter, which may have broadened the candidate target pool while reducing compound-level precision. In addition, the inconsistency between AKT1 mRNA expression in GEPIA and protein-level evidence from HPA and previous reports (60) may reflect post-transcriptional or post-translational regulation. Therefore, AKT1 should be regarded as a mechanistically prioritized candidate hub target rather than a definitively confirmed direct target. Similarly, the docking results should be interpreted as supportive screening evidence rather than a fully benchmarked structure-based validation, because no comprehensive protocol validation against known ligands or re-docking metrics was performed in the current study.

## 5 Conclusion

In summary, this study integrates multiple research methods to suggest that Typhonii Rhizoma (TR) may inhibit colon cancer cell proliferation and migration partly through modulation of the PI3K/AKT signaling pathway under *in vitro* conditions. However, because the current evidence is limited to *in vitro* experiments, further *in vivo* efficacy and safety studies are required before clinical relevance can be inferred.

Future research should focus on (1) evaluating the *in vivo* antitumor efficacy and safety of TR using tumor-bearing mouse models (especially patient-derived xenografts, PDX models); (2) thoroughly screening and identifying its key pharmacodynamic monomers and elucidating their precise molecular targets; and (3) extending validation to more physiologically relevant systems, including 3D spheroids or organoids, tumor–stromal or tumor–immune co-culture models, and tumor-bearing *in vivo* models, to better define the contribution of microenvironmental interactions to the observed effects of TR.

## Data Availability

The datasets presented in this study can be found in online repositories. The names of the repository/repositories and accession number(s) can be found in the article/**Supplementary Material**.
