# Supplementary figures and images for: Integrating network pharmacology, molecular docking, and experimental validation, this study explores the mechanism of action of Typhonii Rhizoma in colon cancer
Source: Front Oncol. 2026 May 7;16:1815506. doi: 10.3389/fonc.2026.1815506 (PMC13189894; doi:10.3389/fonc.2026.1815506)

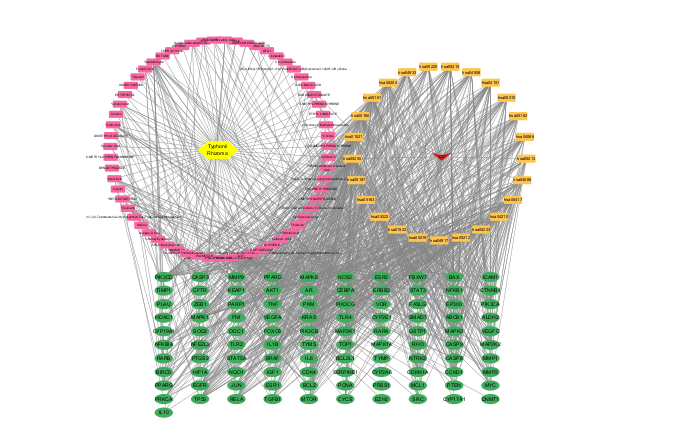

Supplement: Supplementary file 1 [file Image1.png]

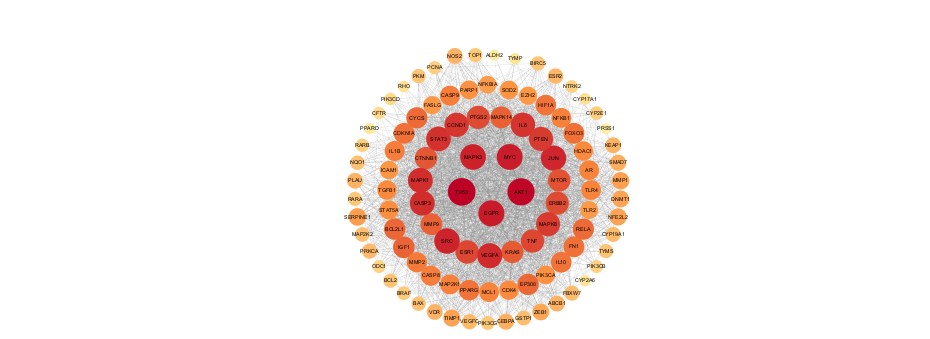

Supplement: Supplementary file 2 [file Image2.png]

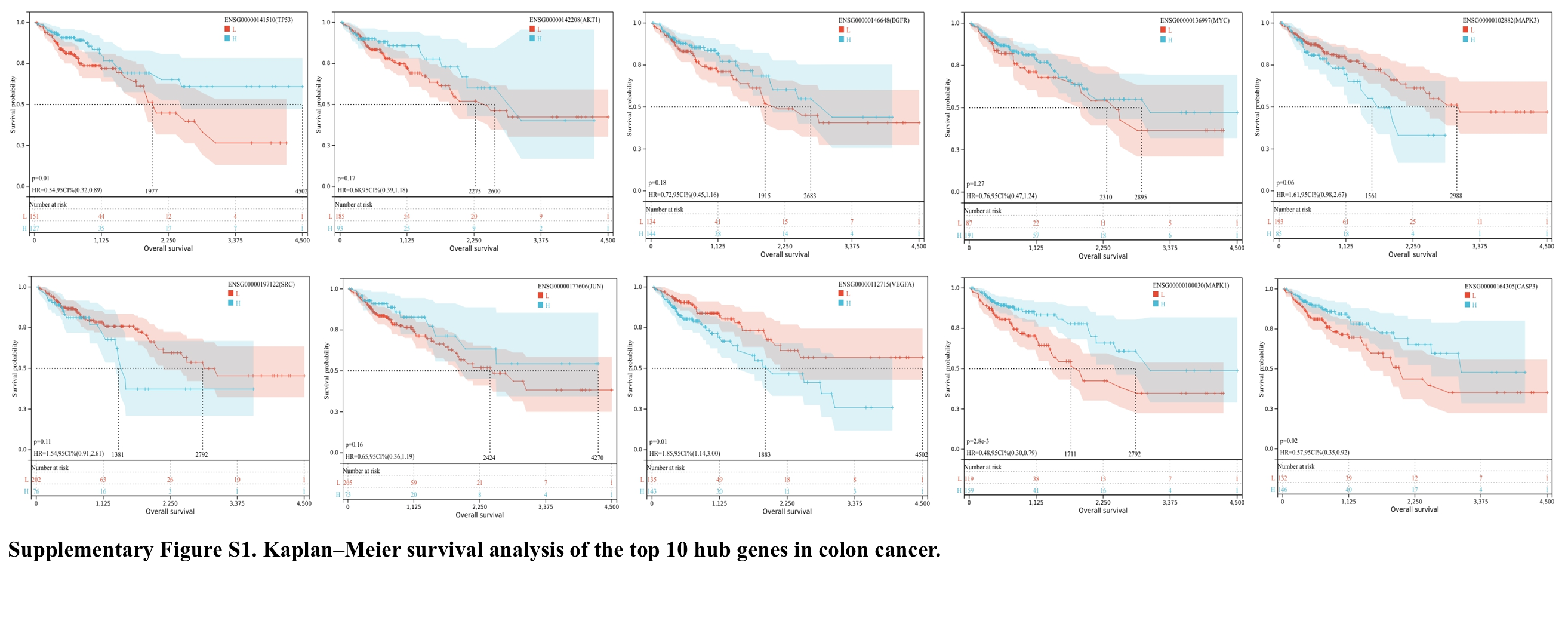

Supplement: Supplementary file 3 [file Image3.png]
